# Supplementary material for: Decision Conflicts in Clinical Care during COVID-19: A Patient Perspective
Source: Healthcare (Basel). 2022 May 31;10(6):1019. doi: 10.3390/healthcare10061019 (PMC9222354; doi:10.3390/healthcare10061019)
Supplement: Supplementary file 1 [file healthcare-10-01019-s001.zip › healthcare-1721893-supplementary.pdf]

Decision Conflicts in Clinical Care During COVID-19: A Patient Perspective

Table S1: Significant differences between disease stage groups at 5-point scale of perception of the (A) decisional modification requirements (p=0.005) and (B) uncertainty (p=0.004); and (C) of education groups decisional modification requirements (p=0.014).

(A)						
Disease Stage	Not at all	A little	Somewhat	A lot	Completely	Total
N/A	15	7	2	4	1	29
Initial Treatment after Diagnosis	116	29	14	11	1	171
Treatment Continuation	98	22	16	8	7	151
Recurrence/Metastasis/Crisis Treatment	56	18	11	20	10	115
Follow up	23	7	7	3	1	41
Total	308	83	50	46	20	507
(B)						
Disease Stage	Not at all	A little	Somewhat	A lot	Completely	Total
N/A	9	7	11	2	1	30
Initial Treatment after Diagnosis	85	44	20	17	5	171
Treatment Continuation	85	30	13	23	4	155
Recurrence/Metastasis/Crisis Treatment	42	27	19	18	9	115
Follow up	20	8	6	3	2	39
Total	241	116	69	63	21	510
(C)						
Education Group	Not at all	A little	Somewhat	A lot	Completely	Total
N/A	8	2	0	2	1	13
Lower education	58	23	1	7	1	90
Intermediate education	144	31	25	15	10	225
Higher education	98	27	24	22	8	179
Total	308	83	50	46	20	507

Table S2: Correlations of the items general uncertainty, uncertainty related to their own treatment, resulting distress and the perception of own risk and requirements for treatment modifications. Pearson correlation, 2-sided significance and numbers of included respondents. ** Significant correlations p<0.01

	General Uncertainty	Own Treatment Uncertainty	Uncertainty Distress	Pandemic Own Risk
Own Treatment Uncertainty	.554**	1	.796**	.275**
	0.000		0.000	0.000
	481	510	496	500
Uncertainty Distress	.557**	.796**	1	.308**
	0.000	0.000		0.000
	474	496	500	490
Pandemic Own Risk	.259**	.275**	.308**	1
	0.000	0.000	0.000	
	483	500	490	511
Own Decisions	.588**	.439**	.417**	.193**
	0.000	0.000	0.000	0.000
	484	479	470	482

Table S3: (A) Correlations of items reflecting the psychological environment of the healthcare professions. Pearson correlation, 2-sided significance and numbers of included respondents. ** Significant correlations p<0.01; (B) Obtained factorial load after PCA-based factorial analysis of items related to individual psychological status; (C) Differences in the distribution of obtained components related to demographic characteristics for psychological status (mean and univariate ANOVA between the groups)

(A)

	Anxiety	Depression	Loneliness	Hope	Stress	Treatment Uncertainty
Depression	.580**					
	0.000					
	527					
Loneliness	.443**	.707**				
	0.000	0.000				
	529	527				
Hope	-0.067	-.299**	-.254**			
	0.122	0.000	0.000			
	526	525	526			
Stress	.538**	.691**	.559**	-.197**		
	0.000	0.000	0.000	0.000		
	526	524	527	523		
Treatment Uncertainty	.465**	.498**	.397**	-.147**	.442**	
	0.000	0.000	0.000	0.001	0.000	
	500	499	510	498	498	
Uncertainty Distress	.475**	.525**	.377**	-.134**	.486**	.796**
	0.000	0.000	0.000	0.003	0.000	0.000
	490	489	500	488	489	496

(B)

Factor components	Factorial load
Depression	0.907
Stress	0.837
Loneliness	0.821
Anxiety	0.730
Hope	-0.368

(C)

Patient subgroups	Component Mean	
Oncology	-0.315	p<0.001
Psychiatry	0.774	
≤ 40 Years	0.741	p<0.001
41-65 Years	-0.107	
>65 Years	-0.394	
Low Education	-0.154	p=0.231
Intermediate Education	-0.018	
High Education	0.093	
Initial Treatment after Diagnosis	-0.276	p<0.001
Treatment Continuation	-0.082	
Recurrence/Metastasis/Crisis Treatment	0.517	
Follow up	-0.206	
Male	-0.223	p<0.001
Female	0.178	

Table S4: Correlations of items reflecting the psychological environment of the healthcare professions. Pearson correlation, 2-sided significance and numbers of included respondents. * Significant correlations p<0.05; ** Significant correlations p<0.01

	Required Treatment Modifications	Treatment Response	Symptoms	Effects/Complications
Treatment Response	.110*			
	0.020			
	446			
Symptoms	.184**	.827**		
	0.000	0.000		
	437	439		
Effects/Complications	.099*	.759**	.786**	
	0.042	0.000	0.000	
	421	423	422	
SARS Additional Risk	.374**	.227**	.314**	.288**
	0.000	0.000	0.000	0.000
	432	428	424	413

Table S5: Prediction of by nominal regression analysis for "Decisional Uncertainty" (A) Likelihood –Quotient-Test for regression parameters; and (B) resulting classification. Inclusion of factor for psychological status

(A)

Effect	-2 Log-Likelihood for reduced model	Likelihood-Quotient-Tests		
		Chi²	dF	Significance
Constant Term	491.117[a]	0.000	0	
Factor Psychological Status	513.671[b]	22.554	4	0.000
Decision Criteria SARS Additional Risk	557.386	66.269	16	0.000
Burden Infection Risk	564.557	73.440	16	0.000
Information Availability Own Provider	549.913	58.796	16	0.000
Education 3 groups	543.249	52.132	12	0.000
Speciality Group	534.373	43.256	4	0.000
Distance Regulations	545.715	54.598	16	0.000
Decision Criteria Side Effects/Complications	517.461[b]	26.344	16	0.049
Decision Criteria Treatment Response	560.341	69.224	16	0.000
Pandemic Own Risk	539.933	48.816	16	0.000

(B)

	Predicted						
Observed	Not at all	A little	Somewhat	A lot	Completely	% Correct	% Correct with neighbor
Not at all	124	14	7	3	0	83.8%	93.2%
A little	31	27	5	7	0	38.6%	90.0%
Somewhat	11	6	19	5	0	46.3%	73.2%
A lot	13	6	5	22	0	47.8%	58.7%
Completely	0	0	0	0	16	100.0%	100.0%
% Total	55.8%	16.5%	11.2%	11.5%	5.0%	64.8%	85.4%
